# Multidendritic sensory neurons in the adult *Drosophila *abdomen: origins, dendritic morphology, and segment- and age-dependent programmed cell death

**DOI:** 10.1186/1749-8104-4-37

**Published:** 2009-10-02

**Authors:** Kohei Shimono, Azusa Fujimoto, Taiichi Tsuyama, Misato Yamamoto-Kochi, Motohiko Sato, Yukako Hattori, Kaoru Sugimura, Tadao Usui, Ken-ichi Kimura, Tadashi Uemura

**Affiliations:** 1 Laboratory of Cell Recognition and Pattern Formation, Graduate School of Biostudies, South Campus Research Building (Building G), Kyoto University, Yoshida Konoe-cho, Sakyo-ku, Kyoto 606-8507, Japan; 2 Laboratory of Neural Development, Graduate School of Biostudies, Kyoto University, Kyoto 606-8501, Japan; 3 Laboratory of Cell Function Dynamics, Advanced Technology Development Group, Brain Science Institute, RIKEN, Wako 351-0198, Japan; 4 Hokkaido University of Education, Sapporo Campus, Sapporo 002-8502, Japan

## Abstract

**Background:**

For the establishment of functional neural circuits that support a wide range of animal behaviors, initial circuits formed in early development have to be reorganized. One way to achieve this is local remodeling of the circuitry hardwiring. To genetically investigate the underlying mechanisms of this remodeling, one model system employs a major group of *Drosophila *multidendritic sensory neurons - the dendritic arborization (da) neurons - which exhibit dramatic dendritic pruning and subsequent growth during metamorphosis. The 15 da neurons are identified in each larval abdominal hemisegment and are classified into four categories - classes I to IV - in order of increasing size of their receptive fields and/or arbor complexity at the mature larval stage. Our knowledge regarding the anatomy and developmental basis of adult da neurons is still fragmentary.

**Results:**

We identified multidendritic neurons in the adult *Drosophila *abdomen, visualized the dendritic arbors of the individual neurons, and traced the origins of those cells back to the larval stage. There were six da neurons in abdominal hemisegment 3 or 4 (A3/4) of the pharate adult and the adult just after eclosion, five of which were persistent larval da neurons. We quantitatively analyzed dendritic arbors of three of the six adult neurons and examined expression in the pharate adult of key transcription factors that result in the larval class-selective dendritic morphologies. The 'baseline design' of A3/4 in the adult was further modified in a segment-dependent and age-dependent manner. One of our notable findings is that a larval class I neuron, ddaE, completed dendritic remodeling in A2 to A4 and then underwent caspase-dependent cell death within 1 week after eclosion, while homologous neurons in A5 and in more posterior segments degenerated at pupal stages. Another finding is that the dendritic arbor of a class IV neuron, v'ada, was immediately reshaped during post-eclosion growth. It exhibited prominent radial-to-lattice transformation in 1-day-old adults, and the resultant lattice-shaped arbor persisted throughout adult life.

**Conclusion:**

Our study provides the basis on which we can investigate the genetic programs controlling dendritic remodeling and programmed cell death of adult neurons, and the life-long maintenance of dendritic arbors.

## Background

The development of the nervous system is not complete at the end of embryogenesis; instead, it involves a series of progressive events. During the postembryonic phase, the nervous system is reorganized at multiple structural levels to strengthen, elaborate, and/or modify already acquired functions, and even add novel ones. This reorganization of initial neural circuits formed during early development is critical to support a wide range of animal behaviors under a variety of environmental contexts [1,2]. One cellular mechanism of this reorganization is the disposal of a subset of early-born neurons and replacement of those cells with later-born ones. Another is 'recycling,' which is accomplished by pruning of axons or dendrites without loss of parental neurons and concomitant or subsequent growth of these processes in spatially distinct patterns [3-5].

Both these cellular mechanisms are observed dramatically in the neural and motor systems of insects that undergo complete metamorphosis (holometabolous insects), which is necessary for the generation of stage-specific behaviors. Nerve and muscle cells that are required for larval behaviors, such as crawling and feeding, must either be replaced or re-specified to allow adult walking, flight, mating, and egg-laying [2,6]. This conversion from larva to adult behaviors is manifested, at least in part, by the remodeling of dendritic arbors. For example, in the early pupae of *Manduca*, an identified motoneuron forms additional dendritic branches, accepting new excitatory synapses from stretch receptors [7]. Therefore, remodeling of stage-specific dendritic patterns is an essential process to realize the proper function of the nervous system.

**Figure 1:**
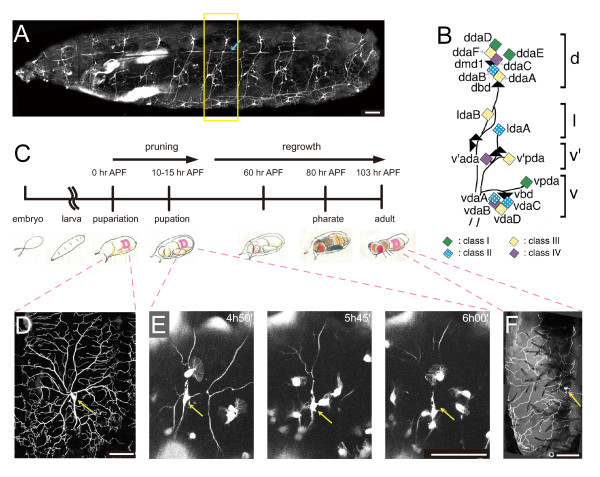
**Larval da neurons and timeline of dendritic remodeling during metamorphosis**. **(A) **A lateral view of a third instar larva that highlights a large subset of dendritic arborization (da) neurons. Bipolar dendrites of dorsal bd (dbd) neurons are marked with blue arrows in this and subsequent figures. Genotype: *UAS-mCD8::GFP*/*UAS-mCD8::GFP*; *C161 UAS-mCD8::GFP*/*TM3 Ser Sb*. Anterior is to the left, and dorsal is at the top throughout this study. **(B) **A diagram of positions of multidendritic (md) neurons in an abdominal hemisegment (A2 to A7) of the *Drosophila *larval peripheral nervous system. Diamonds represent individual da neurons, and triangles represent other types of md neurons. Of the other md types, names of two bipolar dendrite neurons (dbd and vbd) and dmd1 are indicated for simplicity. d, l, v', and v represent dorsal, lateral, ventral prime, and ventral clusters, respectively. Each class of the da neurons is differently colored. Black lines represent fascicles of axons that extend from the neuronal cell bodies. Adapted from [17] with permission from Wiley-Blackwell. **(C-F) **Timeline (C) and representative images (D-F) of dendritic remodeling during metamorphosis. (C) 0 hr after puparium formation (APF) indicates time of pupariation. (D-F) Images of class IV ddaC. The cell bodies are indicated by yellow arrows. (E) We visualized both ddaC and hemocytes and performed time-lapse recordings. Dendritic branches became detached from the cell body (arrow) and disappeared. During the pruning, the hemocytes migrated by generating prominent lamellipodia (see also Additional file 1). (F) ddaC that regenerated its dendritic arbor in the adult tergite. Genotype: (D) *ppk-EGFP*/*ppk-EGFP*, (E) *UAS-EGFP*; *ppk-EGFP*/*pxn-Gal4*, and (F) *FRT19A*/*FRT19A*; *Gal4*^*109(2)80 *^*UAS-mCD8::GFP*/+. Scale bars: 100 μm (A, D-F).

To genetically investigate underlying mechanisms of axon and dendrite remodeling, researchers have used two major model systems, *Drosophila *mushroom body neurons for axons and dendritic arborization (da) neurons for dendrites [4,8,9]. da neurons are born in the embryo, being constituent members of the peripheral nervous system, and generate two-dimensional dendrites underneath the epidermis and on the musculature during late embryonic and larval stages (Figure 1A) [10-12]. The 15 da neurons identified in each abdominal hemisegment are classified into four categories - classes I to IV - in order of increasing size of their receptive fields and/or arbor complexity at the mature larval stage (Figure 1B) [8,12]. In each hemisegment, the three class IV neurons develop the most complicated and expansive arbors with space-filling capability, whereas the three class I neurons generate sparse comb-like dendritic arbors [13,14]. The shaping of these class-specific dendritic arbors is controlled through the expression of a group of transcription factors in the post-mitotic neurons [15-21]. In the sense of physiological function, distinct subclasses appear to be responsible for sensation of proprioception and muscle contractions, which control larval crawling, and that of nociception, which protects larvae from parasitoid wasp attacks [22-24].

It has been reported that a subset of da neurons persists during the pupal transition and that they exhibit large-scale pruning and subsequent regeneration while keeping their axons superficially intact (Figure 1C-F) [25-28]. It is proposed that phagocytic blood cells scavenge neuronal debris and attack intact branches (Figure 1E; Additional file 1) [27]. Two out of the three larval class IV neurons in the hemisegment and one class I neuron, ddaE, are known to be survivors; the dendritic remodeling and migration of ddaE have been described [26,29]. At the molecular level, dendrite-specific pruning requires at least ecdysone signaling, ubiquitin-proteasomes, matrix metalloproteases, the I-κB kinase-related kinase IK2, and a member of the Katanin family of microtubule-severing proteins [29-32]. Nevertheless, our knowledge regarding both the anatomical and molecular bases of the entire remodeling is still fragmentary. Although the soma of persistent larval sensory neurons have been identified [25], the dendritic arbor of each has not been described in the adult, and determination of the one-cell-to-one-cell relationship before and after the remodeling has not been completed either, except for the three neurons described above.

To establish the entire map of adult da neurons, we identified candidates of all survivors in pupae and adults, in both fixed dissected specimens and live whole-mount animals. We also genetically marked individual da neurons and traced the same cells at larval and adult stages. Altogether, our results identified six da neurons in the abdominal hemisegment 3 or 4 (A3/4) of the pharate adult and in the adult just after eclosion; all of these, except for one, were persistent larval da neurons. This 'baseline design' of A3/4 was modified in a segment-dependent and age-dependent manner. Our study provides the anatomical and developmental bases for genetically hunting for mutations that affect remodeling of dendritic arbors or control of the life-long maintenance of differentiated neurons.

## Results

### Overview of peripheral neurons in dissected pharate adults

**Figure 2:**
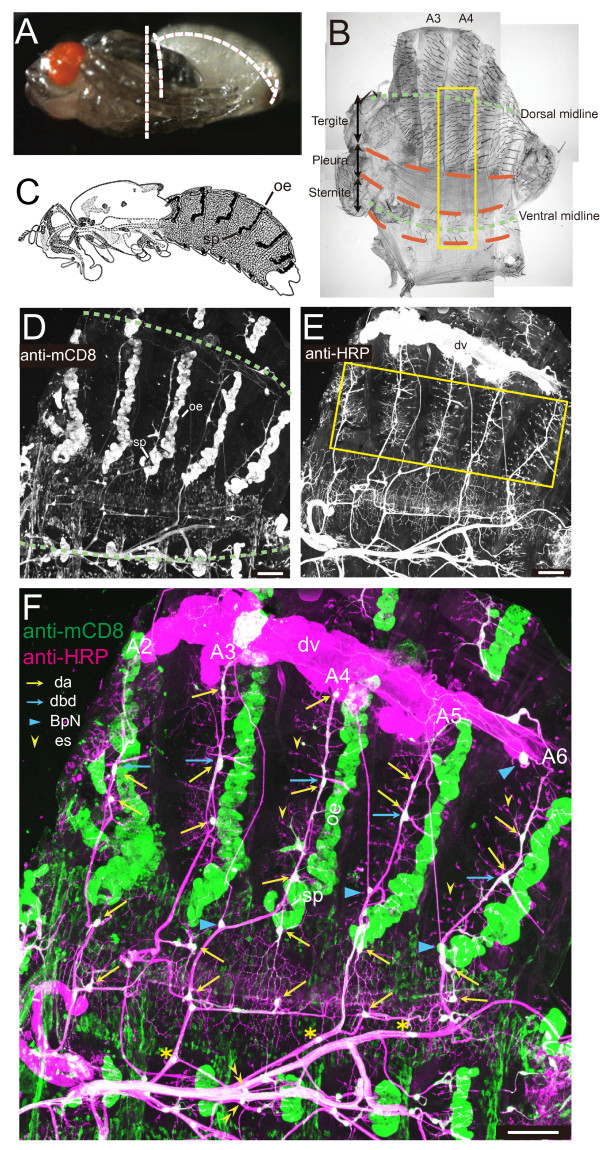
**Distribution of peripheral neurons in the abdomen of the pharate adult**. **(A) **A lateral view of a pharate adult that was taken out of its puparium. Broken lines show how we dissected it. **(B) **A bright-field image of a fillet preparation of the abdomen of a male. Orange broken lines indicate boundaries between tergite, pleura, and sternite; green broken lines here and in (D) indicate dorsal and ventral midlines. The boxed region is highlighted in Figure 3. **(C) **Dark belts in the adult abdomen indicate oenocytes (oe). Adapted from FlyBase. sp, spiracle. **(D-F) **Double labeling of a fillet preparation of a male with anti-mCD8 antibody (D) (green in (F)) and a pan-neuronal marker, anti-HRP antibody (E) (magenta or white in (F)), of genotype *Gal4*^*109(2)80 *^*UAS-mCD8::GFP*/*Gal4*^*109(2)80 *^*UAS-mCD8::GFP*. dv, dorsal vessel (heart). The boxed region in (E) is highlighted in Additional file 4AA. In (F), A2 to A6 indicate numbers of abdominal segments. Yellow arrows point to identified dendritic arborization (da) neurons. Blue arrows indicate dorsal bd neurons (dbds), and blue arrowheads bipolar neurons that innervate the abdominal heart (BpN) [34]. Yellow arrowheads point to representative external sensory (es) neurons, and double yellow arrowheads a pair of v'esB located on both sides of the ventral midline [25]. Asterisks indicate examples of mCD8::GFP-positive cells that were associated with fascicles. Scale bars: 50 μm.

With an expectation of identifying all da neurons in the abdomen of the pharate adult, we dissected animals of a transgenic line in which all larval da neurons are marked (Figure 2A,B; *Gal4*^*109(2)80 *^*UAS-mCD8::GFP*) [11,12] and stained them with anti-mCD8 or anti-green fluorescent protein (anti-GFP) antibody together with a pan-neuronal cell-surface marker, anti-horse radish peroxidase (anti-HRP) antibody [33]. In these fillet preparations, the following three anatomical landmarks were helpful to map candidates of adult da neurons in each segment: a spiracle at the tergite-pleura boundary (sp in Figure 2C,D,F); belts of oenocytes (oe in Figure 2C,D,F); and a bipolar dendrite (bd) neuron in the tergite that had been a dorsal bd (dbd) in larvae (blue arrows in Figure 2F) [26].

**Figure 3:**
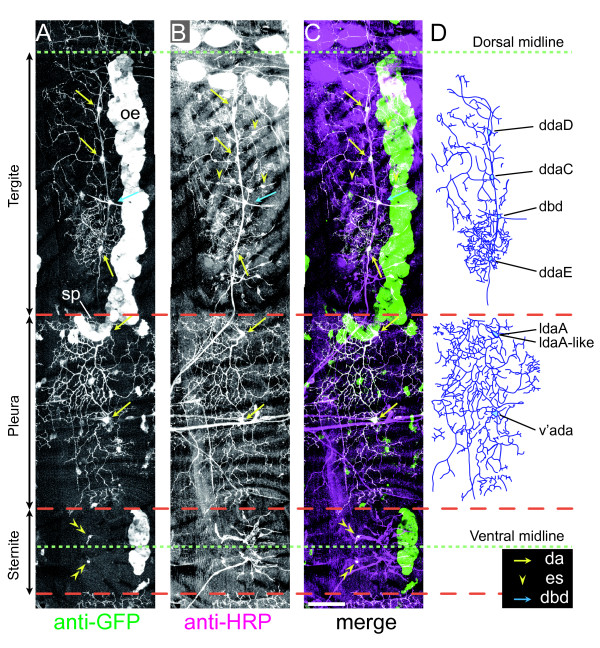
**Overall dendritic arbors of dendritic arborization (da) neurons in hemisegments A3 and A4 of the pharate adult**. *A *fillet preparation of a pharate adult male of genotype *Gal4*^*109(2)80 *^*UAS-mCD8::GFP*/*Gal4*^*109(2)80 *^*UAS-mCD8::GFP *was doubly stained with anti-GFP antibody (A) (green in (C)) and anti-HRP antibody (B) (magenta or white in (C)). **(A-C) **High-power immunohistochemical images of the boxed region in Figure 2B. **(D) **Dendrites, cell bodies, and axons of the da and dorsal bipolar dendrite (dbd) neurons were traced. We traced the origins of individual da neurons back to the larval stage (described later in Figure 4). ldaA and ldaA-like are a pair of closely associated cells (Figure 5G-M). The position of ddaC relative to dbd along the dorsal-ventral axis is not fixed (see text). Cell bodies of the da neurons were located more deeply than their dendrites. The pleura included GFP-positive, HRP-negative cells that had no obvious processes, and they were located more closely to the body surface than cell bodies of the da neurons. Identities of those cells are unknown. Scale bar: 50 μm. es, external sensory; oe, oenocytes; sp, spiracle.

We examined which of mCD8::GFP HRP-double-positive cells extended long and/or complex dendrites, and designated those cells as da neurons at this developmental stage (yellow arrows in Figure 2F; high-power images are shown in Figure 3). In addition to dbd and da neurons, at least three other types of neurons existed in the periphery. First, monodendritic external sensory (es) neurons covered the tergite and the sternite (yellow single and double arrowheads in Figure 2F). Second, a bipolar neuron innervated the abdominal heart (BpN and blue arrowheads in Figure 2F) [34]. Third, a group of neurons was associated with peripheral axon fascicles in the sternite and the pleura (asterisks in Figure 2F).

### Six da neurons in hemisegment A3 or A4 of the pharate adult

We observed the fillet preparations under high magnification, and traced dendrites and cell bodies of mCD8::GFP HRP double-positive cells (Figure 3A-D). Aside from es, dbd, bipolar, and the fascicle-associated neurons, all of the remaining HRP-positive cells also expressed mCD8::GFP; and they appeared to produce much longer and/or far more complex dendrites than es and dbd neurons. This finding suggests that *Gal4*^*109(2)80 *^*UAS-mCD8::GFP *marked all da neurons in pharate adults, as it does in larvae.

**Figure 4:**
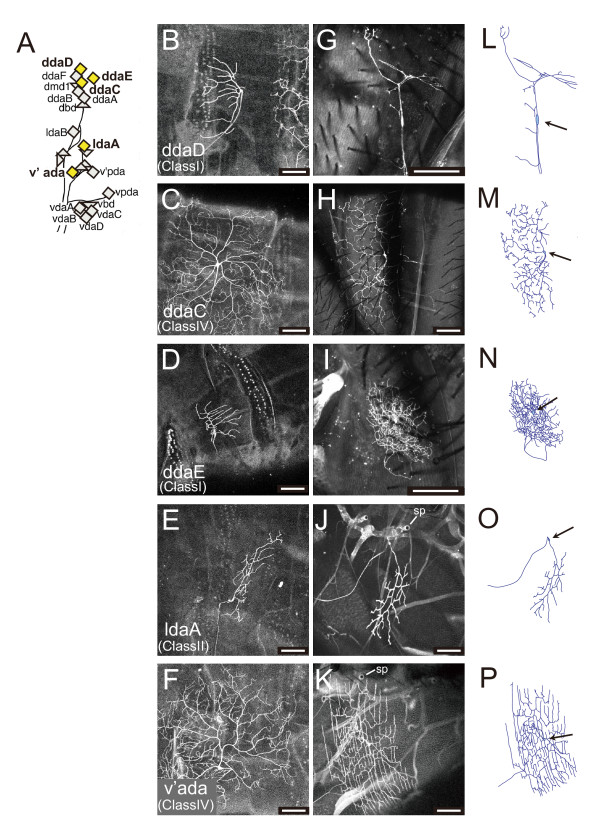
**Relationship between a subset of larval dendritic arborization (da) neurons and the adult ddaD-v'ada**. **(A) **A simplified version of Figure 1B that highlights the five da neurons that survived metamorphosis (yellow diamonds). **(B-K) **Images of MARCM clones identified in third instar larvae (B-H) and of adult da neurons in the same hemisegments of the same animals that had generated the clones (G-K). Pairs of the larval and adult clones are shown side-by-side. Segments that produced clones were A4, A3, A2, A3, and A5 from the top. Genotypes of the clones: *FRT19A*/*FRT19A*; *Gal4*^*109(2)80 *^*UAS-mCD8::GFP*/+ (C-F, H-K) or *Gal4*^*elav[C155] *^*UAS-mCD8::GFP hsflp*/+; *FRTG13 L Sp*/*FRTG13 L Sp *(B, G). Images in (H, J, K) were acquired in 3- to 7-day-old adults, whereas those in (G, I) were of 0- to 12-hour-old adults. sp, spiracle. **(L-P) **Tracings of (G-K). Arrows point to cell bodies. Note that the adult da neurons (G-P) are shown with various scales and that ddaC (H, M) and v'ada (K, P) formed much more expansive arbors than the other neurons. Scale bars: 50 μm.

**Figure 5:**
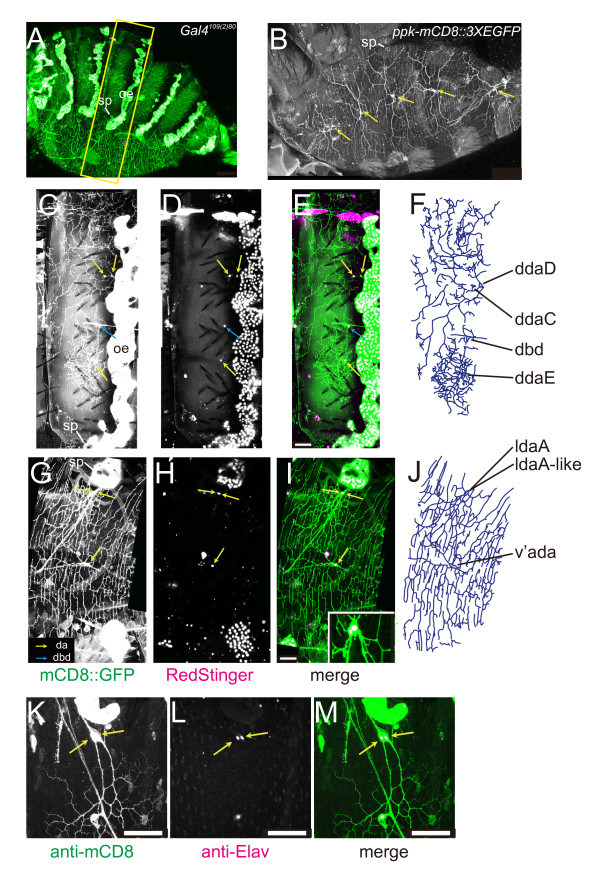
**Dendritic arborization (da) neurons in the adult**. **(A) **A lateral image of a whole-mount abdomen of a 1- to 3-day-old female of genotype *UAS-RedStinger*/+; *Gal4*^*109(2)80 *^*UAS-mCD8::GFP*/+. *RedStinger *encodes a nuclear targeted DsRed. The yellow rectangle indicates A4. oe, oenocyte; sp, spiracle. **(B) **A ventral-lateral image of the pleura of a 3- to 4-day-old male of *ppk-mCD8::3xEGFP*/*ppk-mCD8::3xEGFP*. The pleura was covered with lattice-shaped dendritic arbors of v'ada neurons (yellow arrows), and a couple of branches in each arbor were more strongly labeled than the others. **(C-J) **High-power images of the tergite (C-E) and those of the pleura and the sternite (G-I) in A3/A4 of another whole-mount female of the same genotype as in (A). The channel signals of GFP (C, G) (green in (E, I)) and DsRed (D, H) (magenta or white in (E, I)) are shown. Yellow arrows point to nuclei of da neurons, and blue ones to those of dorsal bipolar dendrite (dbd) neurons. Nuclei of ldaA and ldaA-like are closely associated with each other (G-I; the inset in (I) shows a higher-power image of a different hemisegment). (F, J) Tracings of dendrites and cell bodies in (C) and (G), respectively. **(K-M) **A fillet preparation of a pharate adult female of genotype *Gal4*^*109(2)80 *^*UAS-mCD8::GFP*/*Gal4*^*109(2)80 *^*UAS-mCD8::GFP*. mCD8 staining (K) (green in (M)) and Elav staining (L) (white in (M)), and a merged image (M). This high-power image highlights a pair of nuclei of ldaA and ldaA-like. Scale bars: 50 μm (c-m).

**Figure 6:**
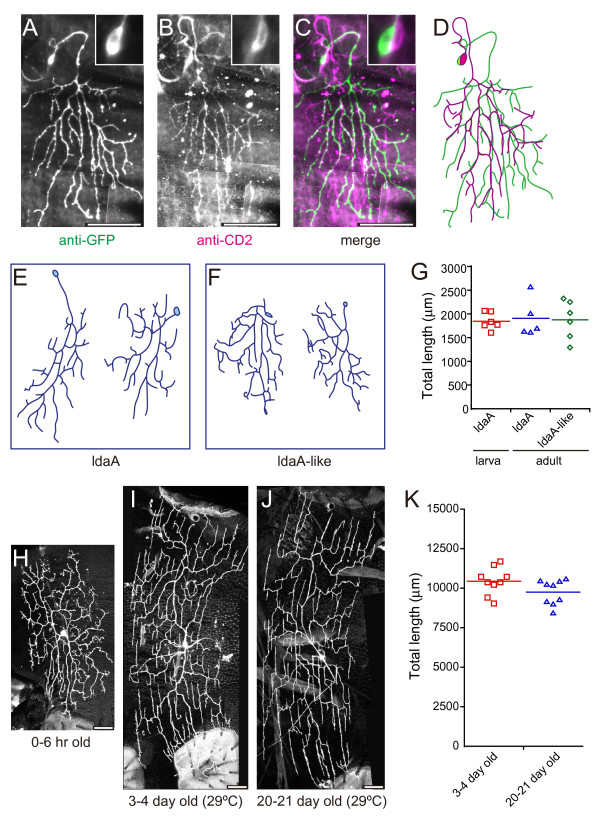
**Morphological features of dendritic arbors of ldaA/ldaA-like and v'ada neurons**. **(A-D) **A pair of ldaA and ldaA-like neurons labeled in different colors by using GFP antibody to visualize the flipout-induced GFP expression (A) (green in (C)) in otherwise CD2-expressing neurons (B) (magenta or white in (C)) in the adult. A merged image (C) and tracings (D) are also shown. Insets indicate high-power images of cell bodies. We did not observe this hemisegment of this animal at a larval stage, so we could not conclude which neuron was derived from larval ldaA. Genotype: *yw hsflp*/*+;UAS-FRT-CD2*,*y*^+-^*FRT-mCD8::GFP*/+; *C*161/*TM2*. **(E-G) **Comparison of arbors of ldaA and ldaA-like neurons. Tracings of representative adult clones of ldaA (E) and ldaA-like (F) in A2 to A5 are shown. These neurons in (E) were designated as ldaA in the adult or simply as 'adult ldaA' because the same hemisegments of individual animals had produced ldaA clones at the larval stage. See the definition of 'ldaA-like' in the text. (G) Plots of total length of dendritic branches of individual larval ldaA (red squares), adult ldaA (blue triangles), and ldaA-like (green diamonds). Horizontal bars indicate means. **(H-K) **MARCM clones of v'ada in A2 to A5 were found, and those adult flies were aged at 29°C. Neurons at 0 to 6 hours (H), 3 to 4 days (I), and 20 to 21 days (J) after eclosion are shown. The clones were generated as described in the legend of Figure 4. (K) Total length of dendritic branches of individual v'ada arbors of A4 and A5 at 3 to 4 days (red squares) and at 20 to 21 days (blue triangles). Horizontal bars indicate means. Scale bars: 50 μm.

On the basis of the neuronal cell-surface staining, we found five da neurons in each of the abdominal hemisegments A3 and A4 (Figure 3D; the number of hemisegments examined was ten for each of A3 and A4). Our cell-marking experiment, which is described in Figure 4, clarified the origin of each neuron; aligned in the dorsal-to-ventral orientation were ddaD (larval class I), ddaC (larval class IV), ddaE (larval class I), ldaA (larval class II), and v'ada (larval class IV). Labeling neuronal nuclei, however, led us to notice that ldaA was associated with another neuron, ldaA-like (Figure 3D; see also Figure 5G-M; explained in Figure 6A-D). Thus, A3 or A4 had six da neurons in total, with three in the tergite and the other three in the pleura. Although it was difficult to distinguish dendritic branches of the individual neurons in this transgenic line, ddaC and v'ada appeared to develop expansive arbors as they did at the larval stage, whereas ddaE formed a bushy but much smaller one. Each ddaD extended possibly a single root dorsally. The dorsal-ventral position of ddaC relative to dbd was not fixed, and ddaC was sometimes located more ventral or attached to dbd. Besides the anti-HRP antibody, we used monoclonal antibody 22C10 as another neuronal marker [35], which broadly labels embryonic and larval peripheral neurons; however, 22C10 stained cell bodies and axons but not dendrites of the da neurons in the pharate adult (data not shown).

**Figure 7:**
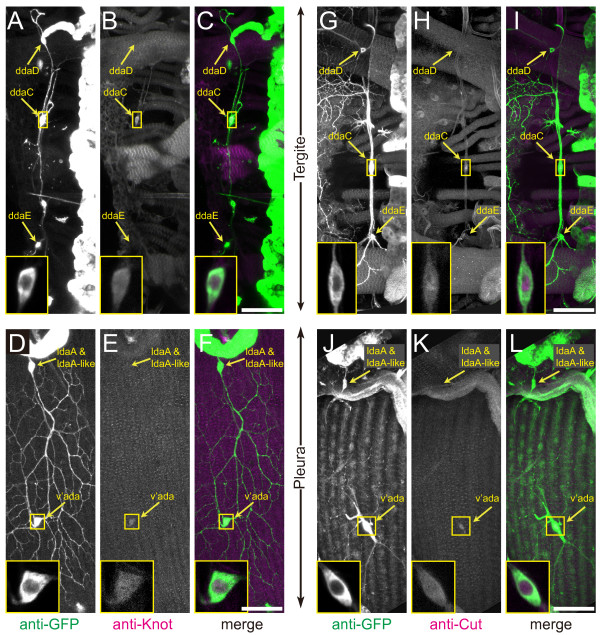
**Expression of shape-controlling transcription factors in pharate adults**. Fillet preparations of pharate adults were doubly stained with anti-GFP antibody and antibody against transcription factors. Yellow arrows point to cell bodies of individual dendritic arborization (da) neurons. Cell bodies of ddaC and v'ada are highlighted in the insets. Genotype: *Gal4*^*109(2)80 *^*UAS-mCD8::GFP*/+. **(A-F) **Neurons stained for GFP (A, D) (green in (C, F)) and for Knot (B, E) (magenta or white in (C, F)). **(G-L) **Neurons stained for GFP (G, J) (green in (I, L)) and for Cut (H, K) (magenta or white in (I, L)). Scale bars: 50 μm.

**Figure 8:**
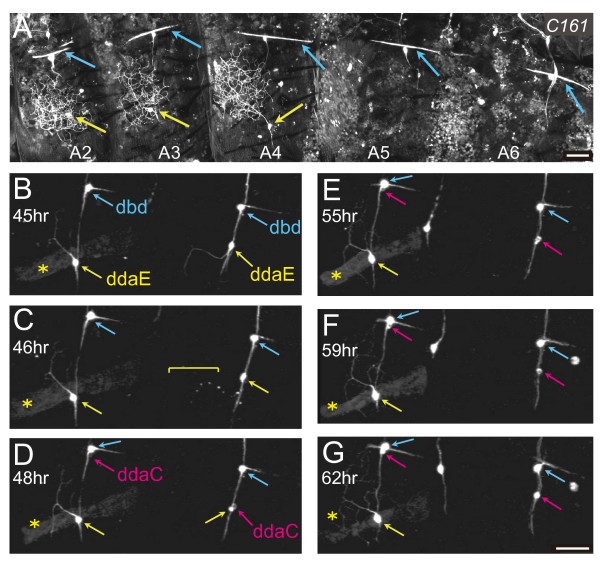
**ddaE in A5 started regeneration of dendrites and then degenerated**. **(A) **A whole-mount of a pharate adult female of genotype *UAS-mCD8::GFP*/+; *C161Gal4*/+. Yellow arrows in A2 to A4 point to cell bodies of ddaE, and blue arrows to dendrites of dorsal bipolar dendrite (dbd) neurons. **(B-G) **Time-lapse recordings of a tergite of A4 and A5 of genotype *UAS-mCD8::GFP*/*UAS-mCD8::GFP*; *C161 UAS-mCD8::GFP*/*TM3 Ser Sb *from 45 h after puparium formation (APF) (B) to 62 h APF (G). Yellow, magenta, and blue arrows indicate ddaE, ddaC, and dbd, respectively. A4 and A5 were identified by the respective presence and absence of a larval persisting muscle (the dorsal internal oblique muscle 3 (DIOM3); yellow asterisks) [43]. A branch of ddaE in A5 was degraded at 46 h APF (yellow bracket in (C)), and then the cell body started condensation (right yellow arrow in (D); see also Additional file 5). Scale bars: 50 μm (A, B-G).

**Figure 9:**
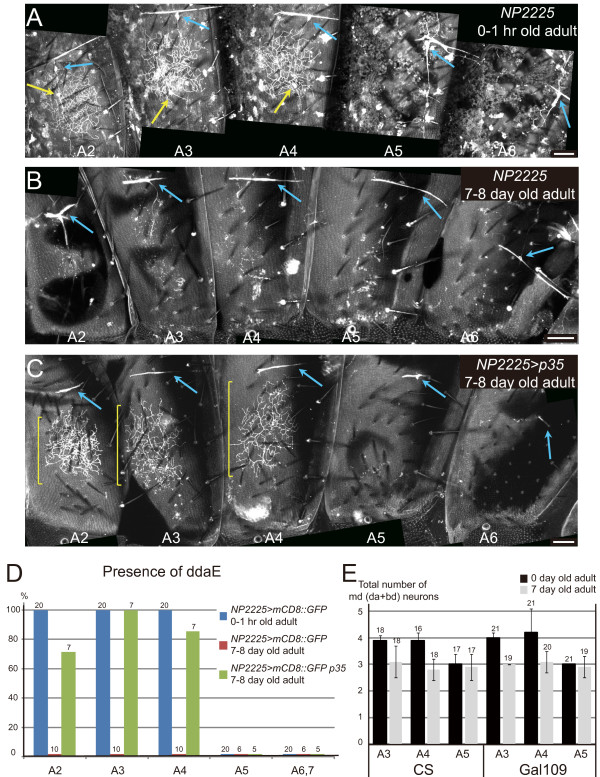
**Almost all ddaE neurons underwent apoptosis within 1 week after eclosion**. **(A-C) **Whole-mounts of a 0- to 1-hour-old female of genotype *NP2225 UAS-mCD8::GFP*/*CyO *(A), a 7- to 8-day-old female of the same genotype (B), and a 7- to 8-day-old female of genotype *NP2225 UAS-mCD8::GFP*/*CyO; UAS-p35/+ *(C). Yellow arrows in (A) point to cell bodies of ddaE in A2 to A4, and blue arrows in (A-C) to those of dorsal bipolar dendrite (dbd) neurons. Yellow brackets in (C) indicate dendritic arbors of surviving ddaE. Scale bars: 50 μm. **(D) **The percentage of each hemisegment that had ddaE. Genotypes and ages of male adults examined are indicated, and the number of each hemisegment observed is given above each bar. Colored short lines just on the x-axis mean that no ddaE signal was found in the hemisegment examined. **(E) **Quantification of the total number of multidendritic (md) neurons (da plus dbd) in each tergite of Canton S (CS) and *Gal4*^*109(2)80 *^*UAS-mCD8::GFP*/*Gal4*^*109(2)80 *^*UAS-mCD8::GFP *(Gal109) in 0- or 7-day-old adults. Adults were dissected and stained for HRP and Elav as in Additional file 4B-FB-F; and the number of md neurons in each tergite was counted. Each value is expressed as mean ± standard deviation. The number of animals examined is indicated above each bar.

With the above information from the fillet preparation in mind, we observed A3 or A4 in live whole-mount pupae of the pan-da marker line and those of marker lines for subsets of larval da neurons (Additional file 2). We found that markers for subsets of larval da neurons were not necessarily reliable tracers to pursue the relationship between larval da neurons and the pharate adult da cells (for example, see Additional file 2GG). We refer to the composition of da neurons in A3 and A4 of the pharate adult as 'the baseline design'. In subsequent sections, we describe origins of individual adult da neurons and their dendritic morphologies (Figures 4, 5 and 6; Additional file 3), expression in the pharate adult of transcription factors that endow larval class-selective dendritic morphologies (Figure 7), and also segment-dependent and age-dependent variations of this design (Figures 8 and 9; Additional file 4).

### da neurons in adults

We then addressed whether the baseline design at the pharate stage persisted in adults or not (Figure 5). Although tracing GFP-labeled dendritic branches in whole-mount adult abdomens was essentially as straightforward as in whole-mount larvae, the feasibility of visualizing branches in the adult specimens depended on sex, age, and whether the tracing was hindered by other intensely labeled cells, such as oenocytes, or not (Figure 5A,B). For example, branches were hardly visible in tergites of A5 and A6/A7 of males due to tanning of the cuticle as they aged (for example, see Figure 4H). Nevertheless, ddaC appeared to form an expansive dendritic arbor; whereas ddaD seemed to have a single dorsally oriented root in A3 to A6 of both sexes, as they did in pharates (Figure 5C-F). To gain stronger membrane signals, some of our images were acquired in transgenic flies that expressed a fusion protein of mCD8 and three tandem copies of enhanced GFP (EGFP; Figure 5B). Co-labeling of membrane-bound GFP and nuclear proteins confirmed the close association of ldaA and ldaA-like (Figure 5I, its inset, and Figure 5K-M).

### Relationship between the larval and adult da neurons

To trace the origins of the adult da neurons back to the larval stage, we labeled individual larval da neurons by the MARCM method [36] and tracked those cells up to the adult stage. More precisely, we first generated clones by using the pan-da driver *Gal4*^*109(2)80 *^or the pan-neuronal driver *C155Gal4 *(*elav-Gal4*), and recorded their cell identities and hemisegments where the clones were located in mature larvae. When the animals became adults, we mounted abdomens and scored all of the marked cells in observable segments as to whether each segment had possessed a clone at the larval stage or not, and attempted to match each of the adult da neurons to any of the larval ones (Figure 4; Table 1).

**Table 1:** MARCM analysis to trace the origins of adult dendritic arborization neurons back to the larval stage.

		**Number of clones in third instar larvae**	**Number of clones in the adults**
d	ddaA (class III)	9	0
	ddaB (class II)	8	0
	ddaC (class IV)	19	19
	ddaD (class I)	11	11^a^
	ddaE (class I)	3	3^a^
	ddaF (class III)	21	0
			
l	ldaA (class II)	6	6
	ldaB (class III)	9	1^b^
			
v'	v'ada (class IV)	10	10
	v'pda (class III)	10	0
			
v	vpda (class I)	6	0
	vdaA (class II)	3	0
	vdaB (class IV)	4	0
	vdaC (class II)	4	1^b^
	vdaD (class III)	2	0

We first generated MARCM clones, and recorded their cell identities and hemisegments where the clones were located in mature larvae. When the animals became adults, we mounted their abdomens and scored all of the marked cells in the recorded hemisegments. We excluded from counting all larvae in which multiple clones were marked in single hemisegments. d, l, v', and v represent dorsal, lateral, ventral prime, and ventral clusters, respectively. ^a^Clones were identified within 12 hours after eclosion. This was partly because *Gal4*^*109(2)80*^-dependent GFP signals were difficult to find in ddaD at later stages, and partly because ddaE executed programmed cell death within 1 week after eclosion. ^b^We interpreted this clone to be ldaA-like that most likely had been derived from a cell other than larval dendritic arborization neurons (see details in the text).

Our analysis revealed one-to-one relationships between four larval da neurons and the four adult ones. When 11 hemisegments that had produced the ddaD (class I) clones in larvae were tracked in adults, it was dorsally located neurons with single dorsally oriented roots that were marked in 11 out of the 11 hemisegments examined; and no such neuron was marked in any of the other hemisegments without ddaD clones (Figure 4B,G). Likewise, all clones of ddaC (class IV) and v'ada (class IV) were matched to neurons with expansive arbors in the tergite and those in the pleura, respectively; moreover, all of the larval ddaE clones were to neurons of small arbors with high branch density (see side-by-side pairs of panels in Figure 4 and numbers of clones examined in Table 1). No other larval da neurons contributed to these three labeled da neurons in the adult (Table 1). These results strongly suggest that each of the four larval da neurons underwent dendritic remodeling to become one of the four adult da neurons. Our results are consistent with reports that MARCM analysis using *C161Gal4 *shows the remodeling of ddaE [26]. Also, observations with the class IV-selective marker *ppk-EGFP *at distinct pupal stages show that ddaC and v'ada survive and they are designated as D neuron and V' neuron, respectively [29] and that there are marked cells in the abdominal body wall of the adult [37].

When we tracked the six hemisegments that had produced ldaA (class II) clones in larvae, labeled in all of them in adults were neurons that were located close to the spiracles, with their dendritic arbors oriented ventrally (Figure 4E,J). In addition to the MARCM analysis, we labeled seven larval ldaA by a flipout-induced GFP expression and found that neurons with the above traits were marked in all of the seven hemisegments. Puzzlingly, we also found five hemisegments in which no larval da neurons had been marked by MARCM; but neurons with cellular locations and arbor shape similar to those of the adult ldaA showed up when the animals became adults. Because labeling of neuronal nuclei showed a pair of neurons to be tightly associated in the vicinity of the spiracle (Figure 5G-M), we speculate that larval ldaA became one of the two neurons in the adult and that the other adult neuron (ldaA-like) was derived from a cell that had not been identified as any of the larval da neurons. We attempted to label a pair of ldaA and ldaA-like in different colors by using the flipout-induced GFP expression in otherwise CD2-expressing neurons in the adult (Figure 6A-D). This labeling method showed that the cell bodies of these two neurons were attached to each other and that their dendritic arbors were substantially overlapped, as we had expected (number of pairs labeled = 3).

### Morphological features of dendritic arbors of the adult da neurons

To understand the morphological features of individual arbors, we analyzed the arbor shape of clones identified in adults. To compare arbors of ldaA neurons with those of ldaA-like ones quantitatively, we traced the arbors of the two neuronal types and measured the total branch length of each type (Figure 6E-G). Dendritic arbors of the two neurons did not differ significantly in terms of total branch length. Intriguingly, v'ada, which had extended dendritic branches in a radial fashion at the pharate stage and just after eclosion, reshaped its arbor into a lattice pattern during post-emergence growth (Figure 6H,I). This radial-to-lattice transformation was recognizable in 1-day-old adults, and most terminal branches tended to be oriented along the dorsal-ventral axis of the body. All of the v'ada clones observed from day 1 onwards elaborated lattice-shaped arbors (Figure 6J). We were able to measure total branch length of the v'ada clones at different adult ages and found that it did not alter during aging (Figure 6K), suggesting that the arbor of v'ada was maintained during adult life. The total branch length of adult v'ada of A4/5 was around 10 mm (Figure 6K), whereas that of the same cell type in the same hemisegments in the wandering third instar was around 15 mm (data not shown).

Compared to ddaC and v'ada arbors, ddaE arbors were smaller in adults (Figure 5F; also compare the scale bar of Figure 4I with that of Figure 4H,K) as they were in larvae [12]. The terminal number of ddaE was remarkably increased after the remodeling, as shown by the fact that each larval ddaE has only about 30 terminals [21], whereas the adult ddaE had over 100 terminals. Apparently, ddaD arbors were the least stereotypic; cell bodies extended single branches dorsally, and they sprouted in a variable fashion (Additional file 3AA). ddaD branches in A3/A4 tended to be longer than those in A5/A6 and to grow along the dorsal vessel.

In addition to the morphological analysis of the clones, we surveyed various *Gal4 *lines for their expression patterns in the adult (Table 2). Some of those lines will be helpful to visualize dendritic arbors at single-cell resolution, to quantify their morphological features, and to analyze physiological functions of the labeled neurons in the future. For example, adult ldaA or ldaA-like was marked by *Gr66aGal4*, which is a fusion of the taste receptor gene *Gr66a *and *Gal4 *(Additional file 3BB) [38], and the same neuron was labeled also by *en-Gal4 *(data not shown; Figure 7D in [39]). Neither *Gr66aGal4 *nor *en-Gal4 *labeled larval ldaA, so we could not conclude whether the labeled neuron in the adult was ldaA or ldaA-like. Gr66a is one of the *Drosophila *caffeine receptors [40,41], and *Gr66aGal4 *labels gustatory receptor neurons that detect aversive chemicals [42].

**Table 2:** Expression patterns of *Gal4 *drivers in the adult *Drosophila *abdomen (A2 to A6).

	**Tergite**			**Pleura**		
**Genotype**	**ddaD**	**ddaC**	**ddaE**	**ldaA, ldaA-like**	**v'ada**	**Reference**
*Gal4 [4-77] UAS-mCD8::GFP*	+	+	+	+	+	[16]
*1003.3Gal4/UAS-mCD8::GFP*	?	+	?	+	+	[22]
*Gal4 *[5-40]/*+; UAS-mCD8::GFP/+*	+	+	+	+	+	[24]
*C155 UAS-mCD8::GFP*	+	+	+	+	+	[60]
*Gal4 [109(2)80] UAS-mCD8::GFP*	+	+	+	+	+	[11]
*NP7028 UAS-mCD8::GFP/CyO*	-	-	-	+	+	[14]
*IG1-2, Gal4 [109], UAS-GFP*	+	+	+	+	+	[14]
*IG1-1 UAS-GFP [S65T] UAS-mCD8::GFP*	+ *	-	+	?	?	[14]
*ppkEGFP*	+	+	-	+	+	[13]
*2-21 UAS-mCD8::GFP*	+*	+	-	-	+	[16]
*Gr66a UAS-mCD8::GFP*	+*	-	-	++^†^	-	[38]
*RluA1-Gal4 UAS-mCD8::GFP*	-	+	+	++	+	[18]
*UAS-mCD8::GFP/+; ppkGal4/+*	+	+	+	+	+	[61,62]
*NP2225 UAS-mCD8::GFP/CyO*	+*	-	++	+	+	[14]
*UAS-mCD8::GFP; C161 UAS-mCD8::GFP/TM3*	+	+	++	++	+	[63]
*Pcol85-Gal4 UAS-mCD8::GFP/CyO Tb*	-	+	-	-	+	[64]

Labeled neurons were identified in whole-mount adults. ++, Strong signal; +, signals were detected with variable intensity among strains; -, signal not detected; ?, difficult to judge due to other close-by signals such as those of oenocytes. *ddaD was labeled only in A5 and A6. ^†^Either ldaA or ldaA-like was labeled. Generally, the entire dendritic arbor of each neuron was more difficult to image just after eclosion than in 3-day or older adults because of signals from other cells such as those of the epidermis.

### Was the dendritic remodeling associated with selective expression of the shape-controlling transcription factors?

It has been shown in larvae that shaping of class-specific dendritic arbors is controlled by a group of transcription factors in the post-mitotic neurons and that some of these factors are expressed in a class-selective or class-differential manner [15-18,20,21]. Knot (Kn)/Collier of the early B cell factor family is expressed selectively in all of the class IV neurons in the embryo, and it is essential for generation of their complicated and expansive arbors [15,17,18]. Its immunohistochemical signal at the mature larval stage was very weak or hardly detected in the class IV neurons (data not shown). Interestingly, in pharate adults Kn expression was revived in both of the two surviving and regrowing class IV neurons (six out of seven ddaC and nine out of nine v'ada examined); on the other hand, Kn expression was detected in no other da neurons at this stage (Figure 7A-F). A homeo-domain protein Cut (Ct) is differentially expressed in distinct subclasses of embryonic and larval da neurons, with the highest level in class III neurons and an intermediate level in class IV neurons [16]. In pharate adults in which larval class III neurons had not been found, we detected only a weak level of Ct expression in the class IV neurons (three out of three ddaC and six out of six v'ada), and none in any other of the da neurons (Figure 7G-L). It should be noted that the Kn or Ct proteins seemed to be distributed in both the nucleus and cytoplasm (see insets of Figure 7), which is in contrast to the almost exclusive nuclear localization in the embryo [15-18]. The simple comb-like dendritic arbor of larval class I neurons (ddaD and ddaE) is endowed by expression of the BTB-zinc finger protein Abrupt (Ab) [20,21]. Ab was expressed in ddaE in mature larvae, whereas the expression was no longer detected in the same cell type when it had remodeled into a highly bushy arbor in the pharate adult (data not shown; number of hemisegments examined = 4). Compared with that in ddaE, Ab expression in ddaD was already weak or hardly detected in mature larvae, and the expression remained off in the pharate adults (data not shown).

### Segment-dependent variations in the composition of da neurons

We found that the A3/A4 baseline design of the da neurons in the tergite was modified in a segment-dependent manner. One obvious difference between segments was that ddaE was invisible in A5 and in more posterior segments in several *Gal4 *lines examined (Figure 8A; see also Figure 9A). Because ddaE is present in all larval A2 to A6 segments, we postulated that ddaE in A5 and A6 had either died during metamorphosis or ceased GFP expression. To distinguish these possibilities, we examined A2 to A6 by staining them with two *Gal4-*independent pan-neuronal markers (Additional file 4). Our results strongly suggest that ddaE was absent in A5 and A6 in the pharate adult. Cell death of ddaE in those segments was verified by our time-lapse recordings (Figure 8B-G; Additional file 5). In A5, a ddaE-like cell started re-growth of its dendrites after the pruning phase (Figure 8B) and then degenerated (Figure 8C). Our staining of neuronal nuclei suggested the composition of da neurons in each segment at the pharate stage (Additional file 4GG).

We could find ddaE neurons in A2 to A4, which had survived pupal development, in 0- to 1-hour adults with complete penetrance (Figure 9A,D); however they became hardly seen by 1 week after eclosion (Figure 9B,D). With the help of the pan-neuronal nuclear marker Elav, we confirmed this loss of ddaE by scoring neuronal nuclei of multidendritic neurons (da plus bipolar dendrite) in dissected adults at the age of 0 or 7 days (Figure 9E). Expression of the caspase inhibitor p35 kept ddaE in A2 to A4 alive, suggesting that ddaE underwent caspase-dependent cell death (Figure 9C,D). In contrast to dendritic arbors of ddaE, those of ddaC, ldaA, and v'ada were visible in transgenic flies over 3 weeks old, even during their entire adult life (data not shown).

## Discussion

### Comparisons with previous reports regarding origins of pupal and adult multidendritic sensory organs

In our study, we identified all multidendritic neurons in the abdomens of the pharate adult and the adult, visualized dendritic arbors of the individual neurons, and attempted to clarify their larval origins. Specifically, we revealed one-to-one relationships between the five larval da neurons and five out of the six da neurons identified in the adult abdomen. Our conclusion regarding ddaC, ddaE, and v'ada agrees with two previous reports that describe those neurons at pupal stages [26,29]. It is reported that larval class I ddaD escapes death and that its dendrites are pruned in prepupa [27]; however, its subsequent fate through to the adult stage was not described. Our result shows that the most dorsally located da neuron with a single dendritic root in the adult abdomen was derived from larval ddaD.

In addition to those studies cited above, embryonically derived sensory neurons are identifiable at pupal and pharate adult stages by using a lineage tracer, although this cell-labeling method does not visualize entire dendritic arbors [25]. That paper reported that class II ldaA is one of the persistent larval sensory neurons, which is consistent with our result. The above paper also describes that class III v'pda is a persistent neuron; however, we found that none of the ten v'pda larval clones persisted in the adult (Table 1) and that it was class IV v'ada in the same v' cluster that survives metamorphosis.

### Segmental differences in the timing of programmed cell death

ddaE neurons in all of the abdominal hemisegments executed programmed cell death (PCD) at pupa to young adult stages, and they showed segmental differences in the execution time window. ddaE in A2 to A4 died within 1 week after eclosion, whereas PCD of ddaE in the more posterior segments took place at the midpupal stage. It should be noted that this segmental difference in the PCD is correlated with the spatiotemporal profile of the breakdown of dorsal internal oblique muscle (DIOM)3, on which ddaE is located (asterisk in Figure 8B-G) [43]. DIOM3 in A5 to A6 dies at 16 to 20 hours after puparium formation (APF), whereas the PCD of DIOM3 in A1 to A4 takes place within 1 day after eclosion. It remains to be studied how the PCD of these two cell types is related, what the physiological significance of the spatiotemporally distinct PCD is and, more fundamentally, what the function of ddaE in pupae and young adults is. We have found strains in which ddaE in A2 to A4 persisted for at least 2 weeks with complete penetrance (KS, KK, and TU, unpublished data). Such strains would be helpful to reveal the underlying mechanisms responsible for the relatively late PCD of ddaE in A2 to A4.

### Pupal and/or adult da neurons provide models whereby we can study intrinsic and extrinsic mechanisms of dendritic morphogenesis and life-long maintenance

In addition to the investigation on the control of PCD described above, pupal and/or adult da neurons provide appropriate models to address other questions regarding arbor-shaping mechanisms. In the sense of a cell-intrinsic mechanism that involves class-selective transcription factors, there were three intriguing findings in pharate adults. First, the class IV neurons (ddaC and v'ada), which had hardly expressed Kn at the mature larval stage, restarted expressing it when they were regenerating complex and expansive arbors. Second, ddaC and v'ada also expressed Ct, as they do in embryonic and larval class IV neurons. Third, class I ddaE transformed its simple comb-like arbor at the larval stage into a highly bushy one without expressing the class I-selective transcription factor Ab. Whether this transcription factor-mediated control plays pivotal roles in dendritic remodeling at pupal stages or not should be tested by either knocking down *kt *or *ct *in the class IV neurons or misexpressing *ab *in class I ddaE neurons selectively during the remodeling phase and then examining the effects on arbor shape. On the other hand, the radial-to-lattice remodeling of the v'ada arbor during post-emergence branch growth implies the possibility that the branch growth is guided by an extrinsic cue(s). One candidate of this cue would be fibers of the lateral tergosternal muscles, which run along the dorsal-ventral axis in the pleura of each segment and become thinner after eclosion [43]. The potential contributions of the intrinsic transcriptional control and the extrinsic cue should be examined, and it should be eventually addressed how critical arbor shape is for ddaE or v'ada to mediate cell type-specific sensory inputs.

Once the lattice arbors of v'ada neurons were generated they persisted throughout adult life, which is 50 days on average. A previous study on class IV neurons showed a pivotal role for the signaling cascade including the Ste-20-related tumor suppressor kinase Hippo (Hpo) in the establishment and subsequent maintenance of dendritic fields during larval development [44,45]. Although dendritic arbors of adult da neurons have to persist for a much longer period than larval development (a total of 5 days), it deserves to be examined whether the larval and adult neurons share the same mechanism to maintain their dendrites or not. The mechanisms that maintain dendritic arbors have been a subject of intense investigation because defective maintenance precedes pathological conditions such as cell death in neurodegeneration [46-48]. One group of gene products responsible for neurodegeneration is associated with mitochondria, and dysfunction of mitochondria in v'ada led to the development of a sparse lattice, which degenerated progressively (A Tsubouchi, TT, and TU, submitted). Therefore, the adult da neuron would provide a novel tractable model for a systematic search for neurodegenerative mutations.

### Long-term *in vivo *time-lapse recordings of pupal da neurons

It has been recently demonstrated in larvae that several organelles and cargos of motor proteins play critical roles in shaping dendritic arbors of da neurons, including Golgi outposts, RNA particles, and endosomes [49-53]. Unfortunately, data collection on organelle behaviors is limited in larval da neurons due to technical difficulties in keeping da neurons in mounted larvae healthy for an hour or even for 10 minutes [54]. Consequently, we have few data on the organelle behaviors that underlie the dynamics of branch elongation, sprouting, and retraction from budding of primary branches to maturation of entire dendritic arbors. One prominent advantage of observing pupal da neurons is that they are accessible to long-term *in vivo *time-lapse recordings. Regrowth and elaboration of the ddaE dendrite can be tracked for up to nearly 2 days ([26]; this study). Simultaneous and long-term tracking of organelles and branches in pupae, and hopefully in adults as well, would allow much more quantitative analysis to determine cause-and-effect relationships between intracellular dynamics and dendrite morphogenesis. One ultimate goal might be to compare all those data on distinct neuronal types such as ldaA and v'ada to understand neuronal type-specific dendritic morphogenesis.

### Conclusion

Our study provides systematic anatomical and developmental descriptions of multidendritic neurons in the adult *Drosophila *abdomen. Knowledge from this study, together with future genetic and physiological studies, will help to reveal the *in vivo *molecular mechanisms governing dendritic remodeling as well as *de novo *dendrite formation, PCD of the adult neuron, and the life-long maintenance of dendritic arbors, and how each of these mechanisms is under the control of neuronal activity.

## Materials and methods

### Drosophila strains

We used the Gal4-UAS system [55] to express most of the transgenes used and to visualize da neurons. Gal4 lines employed in this study to mark da neurons and related references are listed in Table 2. *pxn-Gal4 *[56] was used to visualize hemocytes in pupae. Previously reported UAS fly stocks used were *UAS-GFP [S65T]*, *UAS-mCD8::GFP*, *UAS-myr-mRFP*, and *UAS-Red Stinger *(all provided by the Bloomington Stock Center), *yw hsflp;UAS-FRT-CD2*, *y FRT-mCD8::GFP*/*CyO; TM2/TM6B *[57], and *UAS-p35 *[58]. We generated transgenic flies that expressed a fusion protein of mCD8 and three tandem copies of EGFP as a membrane marker (see Molecular cloning). All fly embryos, larvae, pupae, and adults were grown at 25°C, except for in the experiment shown in Figure 6I-K, in which adults were aged at 29°C [59].

### MARCM analysis and flipout-induced GFP expression

MARCM analyses were performed basically as described previously [36,50]. To label clones with mCD8::GFP, we mated either: *FRT19A*/*FRT19A *females with *hsflp tub-Gal80 FRT19A*/*Y*; *Gal4*^*109(2)80 *^*UAS-mCD8::GFP*/*Gal4*^*109(2)80 *^*UAS-mCD8::GFP *males; *FRT2A*/*FRT2A *females with *hsflp*/*Y*; *Gal4*^*109(2)80 *^*UAS-mCD8::GFP*; *tub-Gal80 FRT2A*/*SM5-TM6B *males; or *FRTG13 L Sp*/*SM6B *males with *Gal4*^*elav[C155] *^*UAS-mCD8::GFP hsflp*/*Gal4*^*elav[C155] *^*UAS-mCD8::GFP hsflp*; *tub-Gal80 FRTG13*/*tub-Gal80 FRTG13 *females. For flipout-induced cell labeling with GFP, *yw hsflp;UAS-FRT-CD2*, *y*^+-^*FRT-mCD8::GFP*/*CyO*; *TM2/TM6B *males were mated with *C161*/*TM6B *females; and the recovered embryos and early larvae were treated with a heat shock (37°C for 30 minutes). Adults with GFP-expressing clones were dissected, fixed, and stained for markers. Detailed genotypes of the animals and clones are described in the legends of individual figures.

### Immunohistochemistry

White prepupae were collected 0 to 0.5 h APF and incubated at 25°C. At appropriate ages, each pupa was placed on double-sticky scotch tape, taken out of its pupal case (puparium), and its thorax pinned down. We covered the pupa with a drop of phosphate-buffered saline, nicked the posterior end of the abdomen, and then cut it in an anterior direction. Adult flies were dissected in a similar way. The opened abdomen was pinned down when necessary, washed in phosphate-buffered saline, and subsequently fixed in phosphate-buffered saline containing 0.1% Triton X-100 (PBT) and 3.7% formaldehyde at room temperature for 30 minutes. After having been washed with PBT, these fillet preparations were stained according to standard protocols with the following primary antibodies: rabbit anti-GFP (Molecular Probes, Eugene, OR, USA), rat anti-mCD8 alpha subunit (Caltag, Burlingname, Californina), rabbit anti-HRP (Jackson ImmunoResearch_West Grove, PA, USA), mouse anti-Elav (Developmental Studies Hybridoma Bank, DSHB), rat anti-Kn [17], rabbit anti-Ab [21], mouse anti-Ct (DSHB), mouse anti-CD2 (Serotec_Oxford, UK), and mouse 22C10 (DSHB). Thoraxes were removed either just after fixation or before mounting the preparations. The abdomens were mounted in FluorSave (Calbiochem, Darmstadt, Germany) and viewed with a laser scanning confocal microscope (Zeiss LSM 510 META, Nikon C1, Leica TCS SPE, or BioRad MRC1024) with a 0.9 or 1.5 μm Z-step. Preservation of peripheral axonal fascicles in the abdomen was sometimes difficult, but improved when a posterior part of the ventral ganglion was left instead of removing the entire thorax. Images of serial optical sections were overlaid to generate each panel in the figures.

### Image acquisition of whole-mount animals and quantitative analysis

Imaging da neurons in whole-mount larvae was done as described earlier [17,50]. For imaging pupal da neurons, each pupa that had been taken out of its puparium was put on a 35-mm glass-bottomed dish (IWAKI, Tokyo, Japan). To prevent desiccation during our time-lapse recordings, we placed a wet filter paper in the dish. After image acquisition, typically with 5% laser power, we kept every pupa at 25°C and confirmed that they survived to the adult stage. Segments observed were identified on the basis of landmarks such as spiracles and larval persisting muscles [43]. To acquire images of da neurons in adults, we removed the heads, wings, and legs of adult flies and mounted the abdomens in 50% glycerol on slides in between spacers made of vinyl tape. All of the images were acquired using laser scanning confocal microscopy. For quantification of dendritic patterns of da neurons, we used Neurocyte software (Kurabo, Osaka, Japan).

### Molecular cloning

We constructed two plasmids, pCaspeR-ppk-hs43-mCD8::3xEGFP and pUAST [mCD8::3xEGFP], and generated trasngenic flies. Briefly, a cDNA fragment encoding mCD8 was amplified from genomic DNA of the pUAST [mCD8::GFP] stock (the Bloomington Stock Center) and ligated with a plasmid that had the open reading frame of 3xEGFP (T Harumoto and TU, unpublished). The fragment encoding mCD8::3xEGFP was recovered and ligated with fragments derived from pCaspeR-ppk-hs43-EGFP ([13]) and pUAST [55] to construct pCaspeR-ppk-hs43- mCD8::3xEGFP and pUAST [mCD8::3xEGFP], respectively.

## Abbreviations

Ab: Abrupt; APF: after puparium formation; Ct: Cut; da: dendritic arborization; dbd: dorsal bipolar dendrite; DIOM: dorsal internal oblique muscle; DSHB: Developmental Studies Hybridoma Bank; es: external sensory; EGFP: enhanced GFP; GFP: green fluorescent protein; HRP: horse radish peroxidase; Kn: Knot; PCD: programmed cell death.

## Competing interests

The authors declare that they have no competing interests.

## Authors' contributions

KS, AF, MS, and KK carried out all immunohistochemical and imaging experiments whose results are shown in the figures. TT, YH, KS, and MY-K performed those experiments at the initial stages of this research. T Us helped with molecular cloning, and T Ue wrote the manuscript.

## Supplementary Material

Additional file 1 **Dendritic pruning and migrating hemocytes**. Class IV ddaC and hemocytes were imaged at 5-minute intervals with a 1.5 μm Z-step between 4 h 50 minutes APF and 6 h APF. Dendritic branches became detached from the cell body and eventually disappeared. The hemocytes were migrating by generating prominent lamellipodia. Genotype: *UAS-EGFP*; *ppk-EGFP*/*pxn-Gal4*. Click here for file

Additional file 2 **Identification of da neurons in live whole-mount pupae**. **(A) **Time-lapse recordings of A4 tergite of a whole-mount pupa of genotype *Gal4*^*109(2)80 *^*UAS-mCD8::GFP*/*Gal4*^*109(2)80 *^*UAS-mCD8::GFP*. By 27 h APF, dendrites had degenerated, and four cells (arrows at 27 h APF) were aligned along the dorsal-ventral axis. Those four cells were identified as indicated. Scale bar: 50 μm. **(B-H) **We also observed whole-mount pupae of the subset markers and addressed whether the specificity of the marker expression persisted in pupae or not. (B-E) The class IV marker *ppk-EGFP *[13] primarily labeled ddaC in the tergite and v'ada in the pleura (not shown) as it did in larvae. Tergite of a whole-mount 60 h APF pupa of genotype *Gal4*^*109(2)80 *^*UAS-mmRFP*/*Gal4*^*109(2)80 *^*UAS- mmRFP*; *ppk-GFP*/*ppk-EGFP*. The channel signal of RFP (B), GFP (C), the merge of these (D), and tracings (E) are shown. (F-H) Example of other markers for 'subsets' of larval da neuons. A3 or A4 tergites of whole-mount 60 h APF pupae of genotype *ppk-Gal4 UAS-mCD8::GFP*/*ppk-Gal4 UAS-mCD8::GFP *(F), which labeled all da neurons, *Gal4*^*477 *^*UAS-mCD8::GFP*/*Gal4*^*477 *^*UAS-mCD8::GFP *(G), which labeled both da and es neurons, and *UAS-mCD8::GFP*/*UAS-mCD8::GFP*; *C161*/*TM3 *(H). In (H), ddaC was weakly labeled and difficult to see in this sample. Expression profiles of various *Gal4 *lines in the adult are summarized in Table 2. Scale bars: 50 μm. Click here for file

Additional file 3 **ddaD and ldaA/ldaA-like in adults**. **(A) **Tracings of dendritic arbors of ddaD clones in A3/A4 (top) and A5/A6 (bottom). Each dendritic branch of ddaD was sometimes difficult to resolve. **(B) ***Gr66aGal4 *visualizes dendritic arbors of ldaA or ldaA-like at single-cell resolution. Pleura of A2 to A6 of *UAS-RedStinger*/*+*; *Gr66aGal4 UAS-mCD8::GFP*/*+ *was imaged through the channel of GFP (green) and DsRed (magenta; overlap with green, white). In each hemisegment, the nucleus of a single neuron (ldaA or lda-like) was labeled (inset; compare with that in Figure 5M). Arrows point to cell bodies. sp: spiracle. Scale bars: 50 μm (A); 100 μm (B). Click here for file

Additional file 4 **Segment-dependent variability in the composition of da neurons in the tergite**. **(A) **A fillet preparation of a pharate adult female was stained with anti-HRP antibody. This image is a high-power view of the boxed region in Figure 2E. Yellow brackets indicate dendritic arbors of ddaE in A2 to A4; ddaE-like cells that formed such bushy dendritic arbors were not found in A5 or A6 (n = 9). Yellow arrowheads indicate cell bodies of representative es neurons, and blue arrows dendrites of dbds. Genotype: *Gal4*^*109(2)80 *^*UAS-mCD8::GFP*/*Gal4*^*109(2)80 *^*UAS-mCD8::GFP*. Scale bar: 50 μm. **(B-F) **Another fillet preparation of the same genotype as in (A), which was doubly stained with anti-mCD8 antibody (B, C-F) and antibody against a pan-neuronal nuclear protein, Elav (C-F). Boxed regions of A2 to A5 in (B) are highlighted in (C-F). (C-F) Three panels of each hemisegment show mCD8 staining (left; green in the right panel), Elav staining (middle; magenta or white in the right panel), and a merged image (right panel). Yellow arrows point to nuclei of da neurons, and blue arrows to those of dbd. Other smaller Elav-positive nuclei are those of es neurons. Typically, the number of the neuronal nuclei of da in the tergite was two in A5 and A6 (F), in comparison to three in A3 and A4 (D, E). Together with the results shown in (A) and Figure 8A, these results strongly suggest that ddaE was absent in A5 and A6 in the pharate adult. The A2 tergite had two, not three, da neurons (C). Our observation using *ppk-EGFP *(a marker of ddaC; Additional file 2CC) and *C161 Gal4 *(a marker of ddaE; Figure 8A) showed that A2 had ddaC and ddaE, but not ddaD. **(G) **Collectively, the composition of da neurons in each segment at the pharate stage is summarized. Plus and minus signs represent the presence and absence of each neuronal type, respectively, and a plus sign with an asterisk means cell death within 1 week after eclosion. Scale bars: 100 μm (B); 50 μm (C-F). Click here for file

Additional file 5 **Degeneration of ddaE in A5**. Time-lapse recording of tergites of A4 and A5 (right). Images were taken every 10 minutes with a 2 μm Z-step between 14 h APF and 62 h APF. A branch of ddaE in A5 was degraded after it started regeneration. Towards the end of the movie, the dorsal internal oblique muscle 1 (DIOM1) is seen at the top (dorsal) in both A4 and A5, whereas DIOM3 was present at a lateral position in only A4. We performed time-lapse recordings of pupae of this genotype a total of four times. Genotype: *UAS-mCD8::GFP*/*UAS-mCD8::GFP*; *C161 UAS-mCD8::GFP*/*TM3 Ser Sb*. Click here for file
