# Supplementary material for: Multidendritic sensory neurons in the adult Drosophila abdomen: origins, dendritic morphology, and segment- and age-dependent programmed cell death
Source: Neural Dev. 2009 Oct 2;4:37. doi: 10.1186/1749-8104-4-37 (PMC2762467; doi:10.1186/1749-8104-4-37)
Supplement: Additional file 2 — Identification of da neurons in live whole-mount pupae. (A) Time-lapse recordings of A4 tergite of a whole-mount pupa of genotype Gal4109(2)80 UAS-mCD8::GFP/Gal4109(2)80 UAS-mCD8::GFP. By 27 h APF, dendrites had degenerated, and four cells (arrows at 27 h APF) were aligned along the dorsal-ventral axis. Those four cells were identified as indicated. Scale bar: 50 μm. (B-H) We also observed whole-mount pupae of the subset markers and addressed whether the specificity of the marker expression persisted in pupae or not. (B-E) The class IV marker ppk-EGFP [13] primarily labeled ddaC in the tergite and v'ada in the pleura (not shown) as it did in larvae. Tergite of a whole-mount 60 h APF pupa of genotype Gal4109(2)80 UAS-mmRFP/Gal4109(2)80 UAS- mmRFP; ppk-GFP/ppk-EGFP. The channel signal of RFP (B), GFP (C), the merge of these (D), and tracings (E) are shown. (F-H) Example of other markers for 'subsets' of larval da neuons. A3 or A4 tergites of whole-mount 60 h APF pupae of genotype ppk-Gal4 UAS-mCD8::GFP/ppk-Gal4 UAS-mCD8::GFP (F), which labeled all da neurons, Gal4477 UAS-mCD8::GFP/Gal4477 UAS-mCD8::GFP (G), which labeled both da and es neurons, and UAS-mCD8::GFP/UAS-mCD8::GFP; C161/TM3 (H). In (H), ddaC was weakly labeled and difficult to see in this sample. Expression profiles of various Gal4 lines in the adult are summarized in Table 2. Scale bars: 50 μm. [file 1749-8104-4-37-S2.PDF]

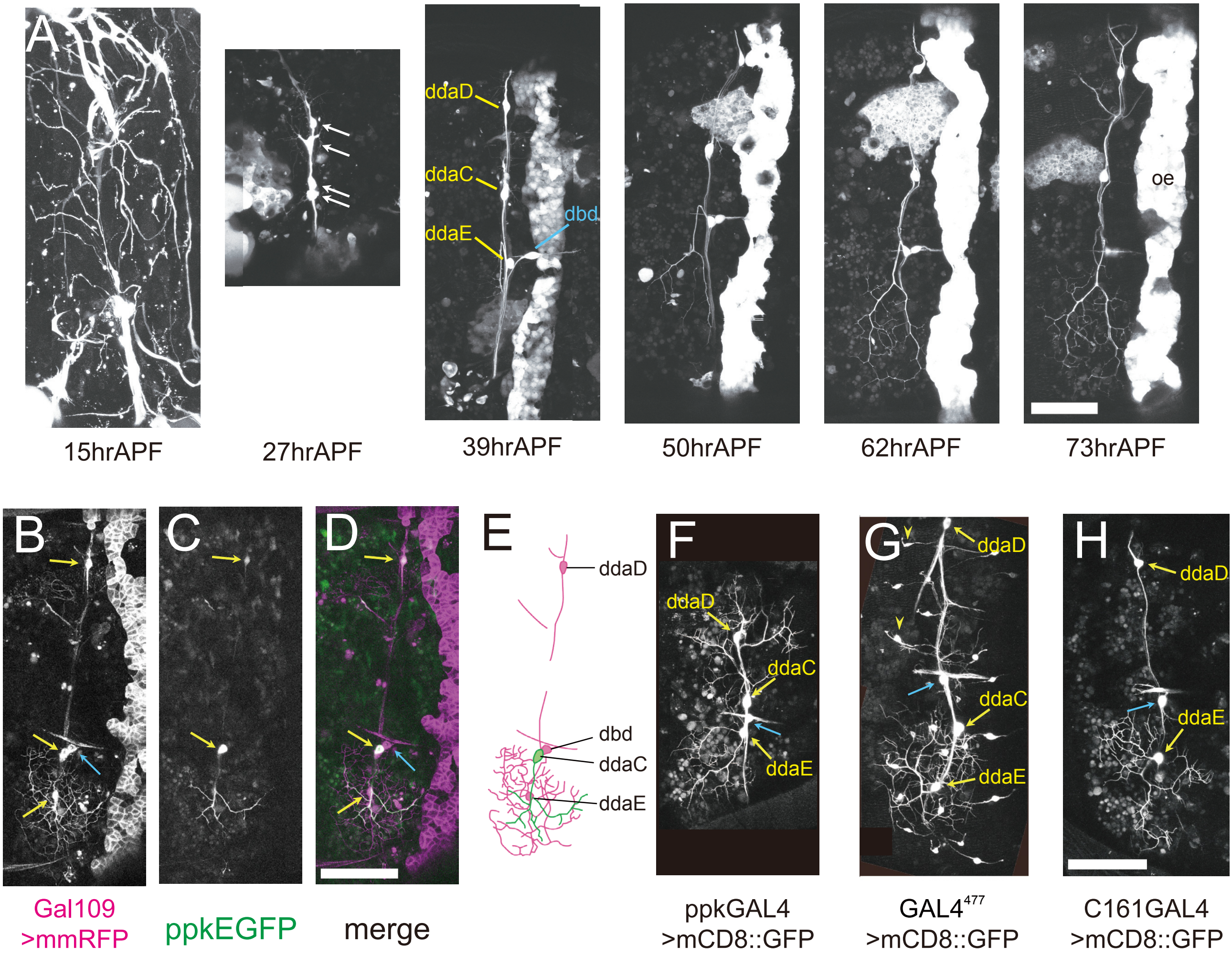
A
15hrAPF
27hrAPF
ddaD
ddaC
ddaE
dbd
39hrAPF
50hrAPF
62hrAPF
oe
73hrAPF
B
Gal109
>mmRFP
C
ppkEGFP
D
merge
E
ddaD
dbd
ddaC
ddaE
F
ddaD
ddaC
ddaE
ppkGAL4
>mCD8::GFP
G
ddaD
ddaC
ddaE
GAL4477
>mCD8::GFP
H
ddaD
ddaE
C161GAL4
>mCD8::GFP
