# Supplementary figures and images for: Multidendritic sensory neurons in the adult Drosophila abdomen: origins, dendritic morphology, and segment- and age-dependent programmed cell death
Source: Neural Dev. 2009 Oct 2;4:37. doi: 10.1186/1749-8104-4-37 (PMC2762467; doi:10.1186/1749-8104-4-37)

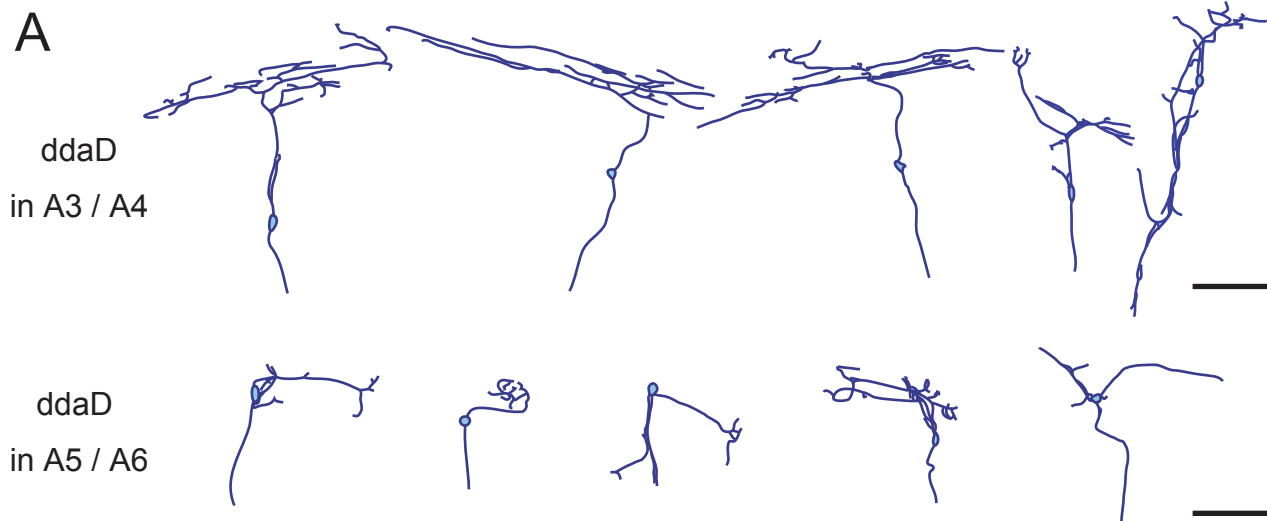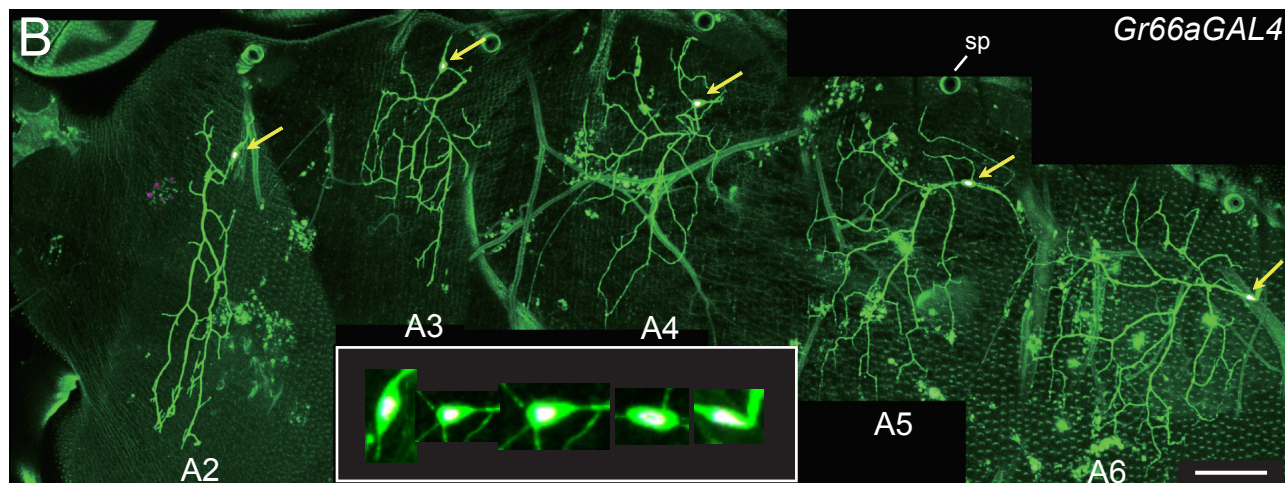

Supplement: Additional file 3 — ddaD and ldaA/ldaA-like in adults. (A) Tracings of dendritic arbors of ddaD clones in A3/A4 (top) and A5/A6 (bottom). Each dendritic branch of ddaD was sometimes difficult to resolve. (B) Gr66aGal4 visualizes dendritic arbors of ldaA or ldaA-like at single-cell resolution. Pleura of A2 to A6 of UAS-RedStinger/+; Gr66aGal4 UAS-mCD8::GFP/+ was imaged through the channel of GFP (green) and DsRed (magenta; overlap with green, white). In each hemisegment, the nucleus of a single neuron (ldaA or lda-like) was labeled (inset; compare with that in Figure 5M). Arrows point to cell bodies. sp: spiracle. Scale bars: 50 μm (A); 100 μm (B). [file 1749-8104-4-37-S3.PDF]

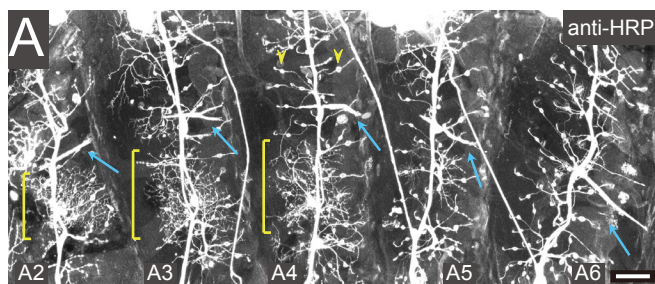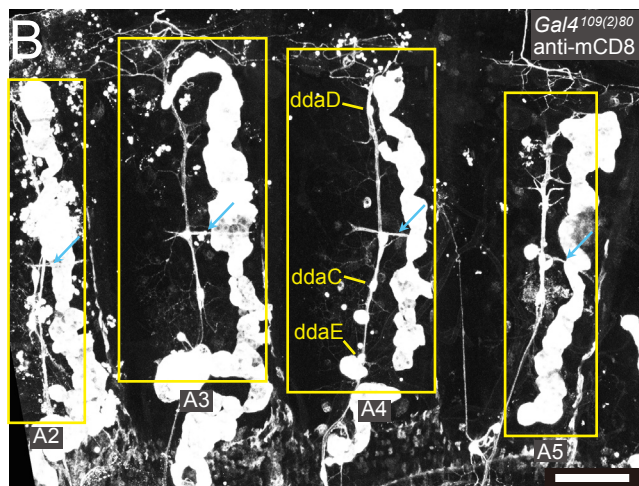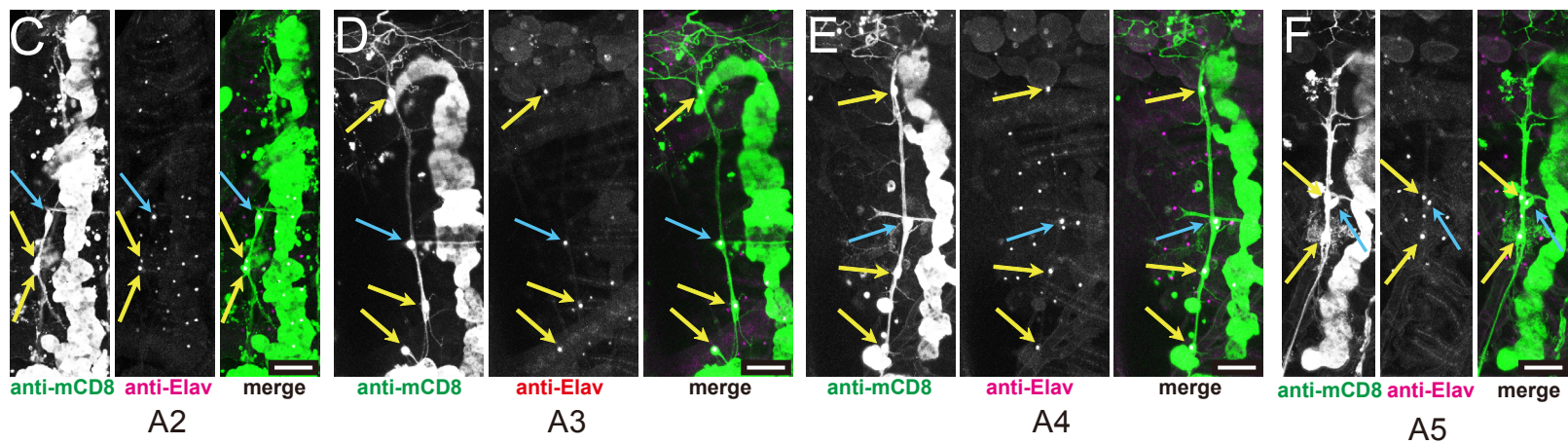

**G**

|         |  | A2        | A3 | A4 | A5 | A6 |
|---------|--|-----------|----|----|----|----|
| Tergite |  | ddaD      | -  | +  | +  | +  |
|         |  | ddaC      | +  | +  | +  | +  |
|         |  | ddaE      | +* | +* | +* | -  |
| Pleura  |  | IdaA      | +  | +  | +  | +  |
|         |  | IdaA-like | +  | +  | +  | +  |
|         |  | v'ada     | +  | +  | +  | +  |
|         |  |           |    |    |    |    |

Supplement: Additional file 4 — Segment-dependent variability in the composition of da neurons in the tergite. (A) A fillet preparation of a pharate adult female was stained with anti-HRP antibody. This image is a high-power view of the boxed region in Figure 2E. Yellow brackets indicate dendritic arbors of ddaE in A2 to A4; ddaE-like cells that formed such bushy dendritic arbors were not found in A5 or A6 (n = 9). Yellow arrowheads indicate cell bodies of representative es neurons, and blue arrows dendrites of dbds. Genotype: Gal4109(2)80 UAS-mCD8::GFP/Gal4109(2)80 UAS-mCD8::GFP. Scale bar: 50 μm. (B-F) Another fillet preparation of the same genotype as in (A), which was doubly stained with anti-mCD8 antibody (B, C-F) and antibody against a pan-neuronal nuclear protein, Elav (C-F). Boxed regions of A2 to A5 in (B) are highlighted in (C-F). (C-F) Three panels of each hemisegment show mCD8 staining (left; green in the right panel), Elav staining (middle; magenta or white in the right panel), and a merged image (right panel). Yellow arrows point to nuclei of da neurons, and blue arrows to those of dbd. Other smaller Elav-positive nuclei are those of es neurons. Typically, the number of the neuronal nuclei of da in the tergite was two in A5 and A6 (F), in comparison to three in A3 and A4 (D, E). Together with the results shown in (A) and Figure 8A, these results strongly suggest that ddaE was absent in A5 and A6 in the pharate adult. The A2 tergite had two, not three, da neurons (C). Our observation using ppk-EGFP (a marker of ddaC; Additional file 2CC) and C161 Gal4 (a marker of ddaE; Figure 8A) showed that A2 had ddaC and ddaE, but not ddaD. (G) Collectively, the composition of da neurons in each segment at the pharate stage is summarized. Plus and minus signs represent the presence and absence of each neuronal type, respectively, and a plus sign with an asterisk means cell death within 1 week after eclosion. Scale bars: 100 μm (B); 50 μm (C-F). [file 1749-8104-4-37-S4.PDF]
